# QTL analysis and dissection of panicle components in rice using advanced backcross populations derived from *Oryza Sativa* cultivars HR1128 and ‘Nipponbare’

**DOI:** 10.1371/journal.pone.0175692

**Published:** 2017-04-19

**Authors:** Zhizhong Sun, Xiaoling Yin, Jia Ding, Dong Yu, Miao Hu, Xuewu Sun, Yanning Tan, Xiabing Sheng, Ling Liu, Yi Mo, Ning Ouyang, Beibei Jiang, Guilong Yuan, Meijuan Duan, Dingyang Yuan, Jun Fang

**Affiliations:** 1 College of Bioscience and Biotechnology, Hunan Agricultural University, Changsha, Hunan, China; 2 State Key Laboratory of Hybrid Rice, Hunan Hybrid Rice Research Center, Changsha, Hunan, China; 3 Hunan Academy of Agricultural Sciences, Changsha, Hunan, China; 4 Long Ping Branch, Graduate School of Hunan University, Changsha, Hunan, China; International Rice Research Institute, PHILIPPINES

## Abstract

Panicle traits are among the most important agronomic characters which directly relate to yield in rice. Grain number (GN), panicle length (PL), primary branch number (PBN), and secondary branch number (SBN) are the major components of rice panicle structure, and are all controlled by quantitative trait loci (QTLs). In our research, four advanced backcross overlapping populations (BIL152, BIL196a, BIL196b, and BIL196b-156) carrying introgressed segments from chromosome 6 were derived from an *indica*/*japonica* cross that used the super-hybrid rice restorer line HR1128 and the international sequenced japonica cultivar ‘Nipponbare’ as the donor and recurrent parents, respectively. The four panicle traits, GN, PL, PBN, and SBN, were evaluated for QTL effects using the inclusive composite interval mapping (ICIM) method in populations over two years at two sites. Results showed that a total of twelve QTLs for GN, PL, PBN, and SBN were detected on chromosome 6. Based on marker loci physical positions, the QTLs were found to be tightly linked to three important chromosomal intervals described as RM7213 to RM19962, RM20000 to RM20210, and RM412 to RM20595. Three QTLs identified in this study, *PL6-5*, *PBN6-1*, and *PBN6-2*, were found to be novel compared with previous studies. A major QTL (*PL6-5*) for panicle length was detected in all four populations at two locations, and its position was narrowed down to a 1.3Mb region on chromosome 6. Near isogenic lines (NILs) carrying *PL6-5* will be developed for fine mapping of the QTL, and our results will provide referable information for gene excavation of panicle components in rice.

## Introduction

In rice, panicle architecture is not only the most important component of plant type, but is also a vital factor for improving grain yield. Grain number (GN), panicle length (PL), primary branch number (PBN), and secondary branch number (SBN) are the major components of rice panicle structure. Many studies have shown that panicle structure and the component traits are all typical and complex quantitative traits which are affected by genes and environment, and that together they determine the design of plant architecture and yield in rice [1–3].

With the development of molecular markers and rice mapping populations, many quantitative trait loci (QTL) for panicle structure components have been reported [4–11] and some have been fine mapped [12–16]. Except the many mapped QTLs for panicle structure, some of those with large effects defined major genes that have been cloned. In 2005, the first gene for grain number, *Gn1a*, was cloned and found to be a major QTL that encodes a cytokinin oxidase gene, and this gave the first indication that endogenous hormone levels could regulate grain yield in rice. Recently, studies of the zinc finger transcription factor *DST* have allowed a further understanding into the formation of spikelet number per panicle, which is regulated through *Gn1a* [17, 18]. The *IPA1* QTL is encoded by *OsSPL14* (SOUAMOSA PROMOTER BINDING PROTEIN-LIKE 14) and was found to be regulated by microRNA (miRNA) OsmiR156 *in vivo*. Further research demonstrated that a point mutation in *OsSPL14* disrupts OsmiR156-directed regulation of *OsSPL14*, generating an 'ideal' rice plant with reduced tiller number, increased lodging resistance, and enhanced grain yield. In addition, other studies showed that *IPA1* could not only direct binding to the promoter of the negative regulatory factor gene *OsTB1*, thus suppressing tillering in rice, but also affected plant height and panicle length, which impacted yield through positive control of the important plant architecture gene *DEP1* [19–21]. DNA sequence analysis of the aberrant *panicle organization 1* (*apo1*) mutant indicated that the gene encodes an F-box protein which is orthologous to the regulatory factor UFO of class-B genes in *Arabidopsis thaliana*, and showed that *APO1* positively controls spikelet number by suppressing the precocious conversion of the inflorescence [22, 23]. The identification and cloning of a novel rice mutant gene, *short panicle1* (*sp1*), showed that this gene encodes a putative transporter that belongs to the peptide transporter (PTR) family, which supported the previous finding that SP1 contains a conserved PTR2 domain consisting of 12 transmembrane domains, and that the SP1-GFP fusion protein is localized to the plasma membrane [24]. The *lax2* mutant is similar to *lax panicle1* (*lax1*) in that it lacks an apical meristem (AM) in most of the lateral branches of the panicle and has a reduced number of AMs at the vegetative stage. The *lax1 lax2* double mutant synergistically enhances the reduced-branching phenotype, indicating the presence of multiple pathways for branching. *Lax2* encodes a nuclear protein that contains a plant-specific conserved domain and physically interacts with LAX1 [25, 26].

In this study, four advanced backcross populations derived from an *indica/japonica* cross that used the super-hybrid rice restorer line HR1128 and the international sequenced japonica cultivar ‘Nipponbare’ as the donor and recurrent parents, respectively, were developed for QTL analysis of rice panicle structure which included the traits GN, PL, PBN, and SBN. We mapped twelve QTLs for panicle components, and among these, three QTLs contained *PL6-5*, *PBN6-1*, and *PBN6-2* were found to be novel. A major QTL (*PL6-5*) for panicle length was detected on the long arm of chromosome 6 and was narrowed down to a physical region of 1.3 Mb. These results will provide referable information for gene excavation of panicle components in rice, and near isogenic lines (NILs) carrying *PL6-5* will then be developed for the fine mapping of panicle length.

## Materials and methods

### Population development

**Fig 1:**
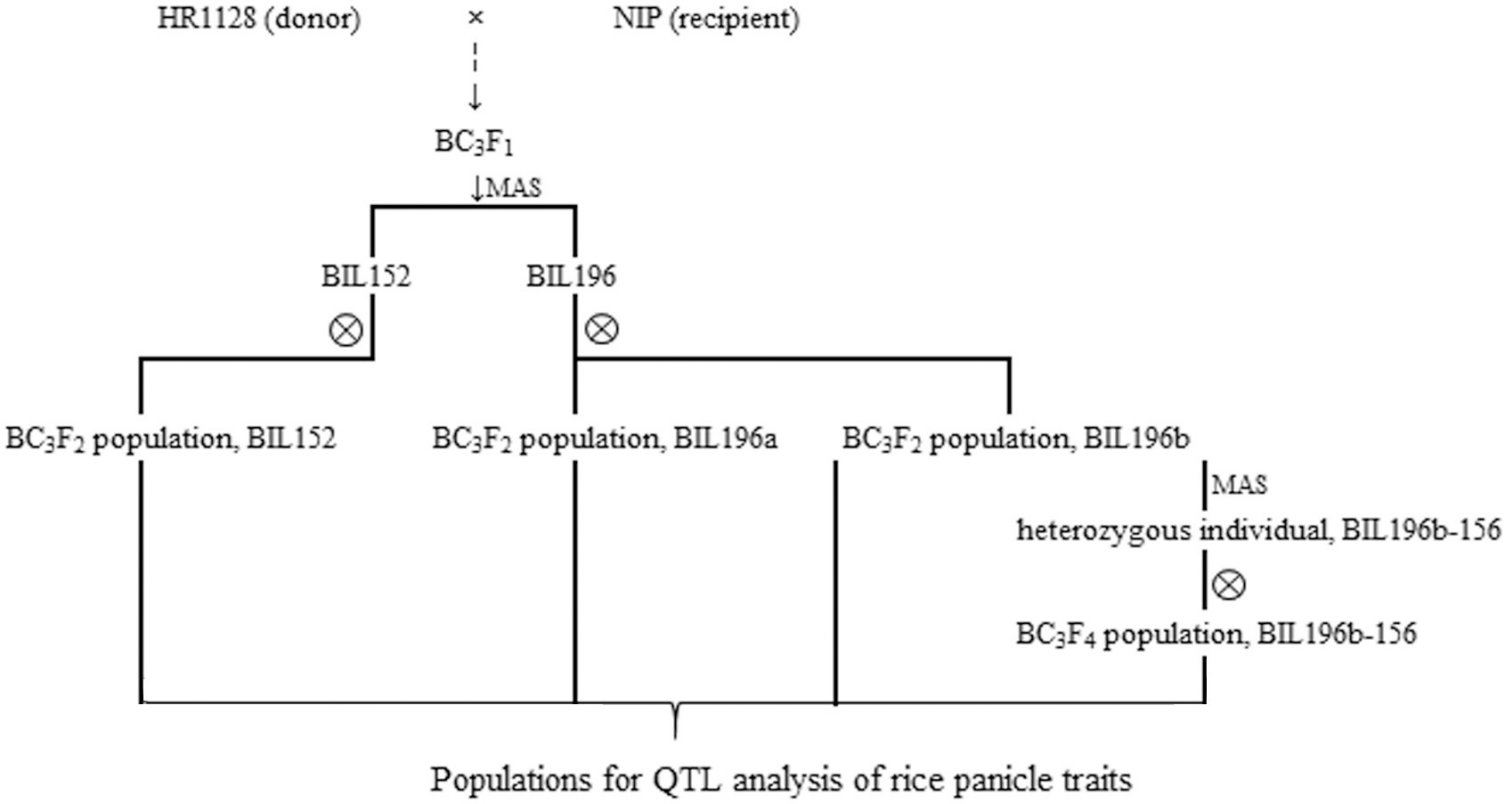
Flow chart of population development for QTL analysis.

**Fig 2:**
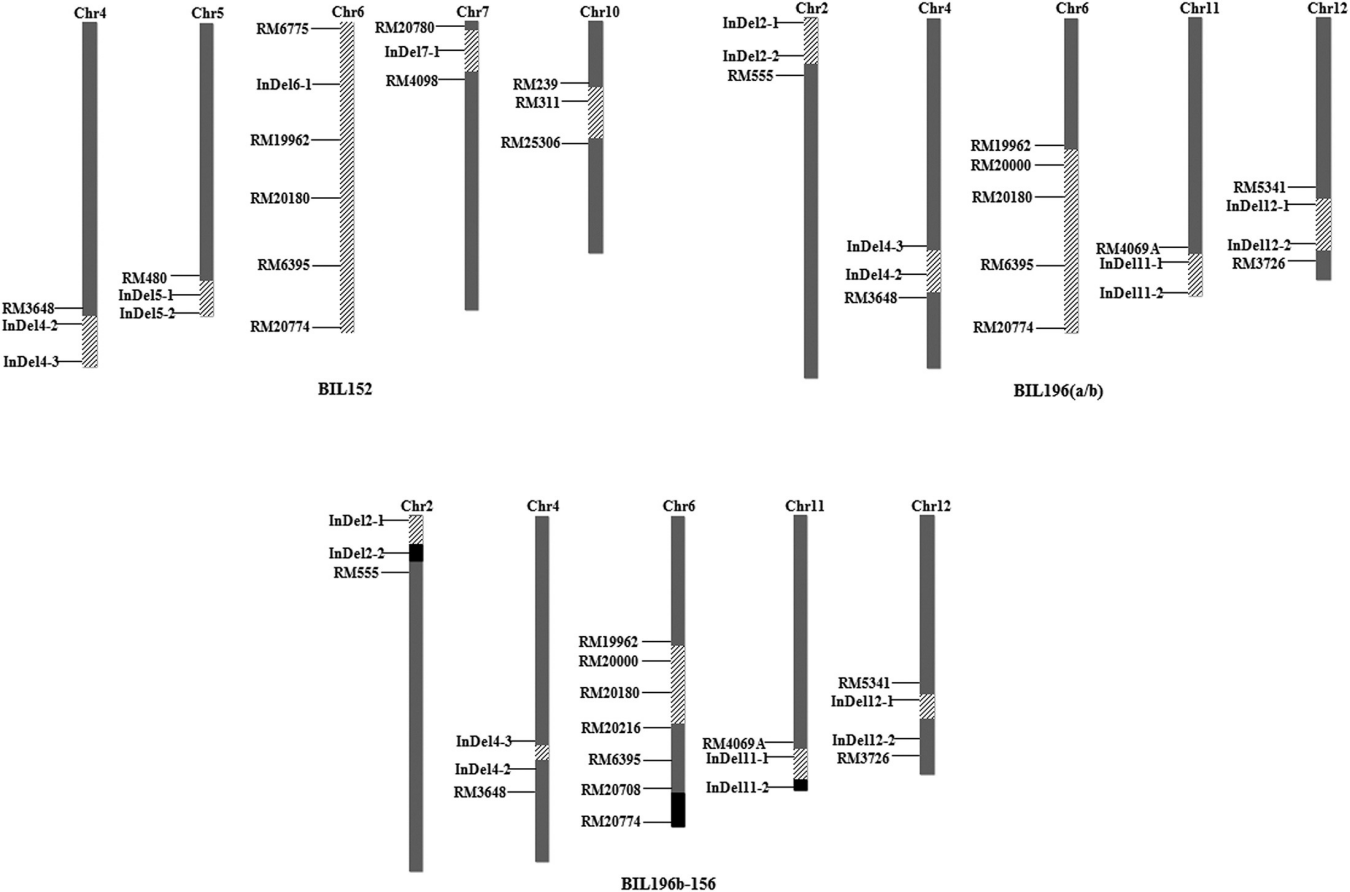
Graphic genotype of the selected advanced backcross individuals. The grey and black regions of the chromosomes indicate the homozygous segments from Nipponbare and HR1128 respectively. The hatched regions of the chromosomes indicate the heterozygous areas.

In this study, four advanced backcross populations were selected for QTL mapping of panicle components in rice (Fig 1). The donor parent was an *indica* super-hybrid rice restorer line, HR1128 [27], which was previously chosen to be a high-yield QTL donor for high throughput genetic analysis [28]. The recurrent parent was the *japonica* cultivar ‘Nipponbare’, the first rice cultivar to be genome sequenced [29], which was previously used as a common recurrent parent for population development and multiple QTL analyses [30–32]. Based on previous results, we chose a single plant carrying the target fragment on chromosome 6 to backcross with the recurrent parent continuously. Following this, the BC_3_F_1_ plant BIL196, and BIL152, which was heterozygous in this region, were selected for self-pollination, which gave 331 and 200 BC_3_F_2_ plants, respectively, as the mapping population. A second population, named BIL196b-156 (BC_3_F_4_, 384 plants), was derived from self-pollination of a BIL196b progeny plant called 156, which carried fewer heterozygous segments on chromosome 6 (Fig 2).

### Field experiments and phenotypic evaluation

The population made up of 331 BC_3_F_2_ plants generated from BIL196 was divided into two groups, BIL196a and BIL196b. BIL196a, consisting of 199 BC_3_F_2_ plants, was planted at Sanya in Hainan province in the winter of 2014, while BIL196b, consisting of 132 BC_3_F_2_ plants, was grown at Changsha in Hunan province in the summer of 2014. Another population consisting of 200 individuals derived from the heterozygous plant BIL152 was cultivated at Sanya adjacent to population BIL196a, while the final population BIL196b-156 was grown at Sanya in Hainan province in the winter of 2015. The donor and recurrent parents were included as controls with three replicates of each. All plant material was cultivated using standard field management practices. A number of panicle characters including grain number (GN), panicle length (PL), primary branch number (PBN), and secondary branch number (SBN) were measured in the two parental lines and the four populations. The survey standard was the same as that of Duan et al [28].

### DNA extraction and marker genotyping

Genomic DNA was extracted from small tissue samples from the parental lines and all populations at the seedling stage using the CTAB method [33]. In this study, we used SSR markers which were obtained from the Gramene database (http://www.gramene.org/) for linkage map construction and QTL mapping. Marker genotyping and assays were performed using 8% denaturing polyacrylamide gels as described by Wu and Tanksley [34].

### Linkage map construction and QTL analysis

Genetic linkage maps of all populations were constructed with the program Mapmaker/Exp3.0 [35]. QTL analysis was performed using the inclusive composite interval mapping (ICIM) method in IciMapping, version 4.0, based on a stepwise linear regression model [36]. QTL mapping in the present experiment was carried out by calculating the threshold logarithm of odds difference (LOD) for each trait by performing a test with 1,000 permutations. The threshold of LOD values was 2.5. QTL were named according to McCouch et al. [37] and the QTL mapping results were comprehensively compared to the OGRO database [38], the Q-TARO database [39] and Gramene (http://archive.gramene.org/qtl/). The statistical significances for the population and parental trait data were calculated with Microsoft Excel. Phenotypic correlation coefficients were calculated using SPSS13.0 software (SPSS Inc., Chicago, IL).

## Results

### Phenotypic data analysis of parental lines and populations

In our research, the four panicle-related traits, grain number (GN), panicle length (PL), primary branch number (PBN), and secondary branch number (SBN), were investigated in four populations (BIL152, BIL196a, BIL196b, and BIL196b-156) and the parental lines at SY (SanYa) in 2014 and CS (ChangSha) in 2015.

**Fig 3:**
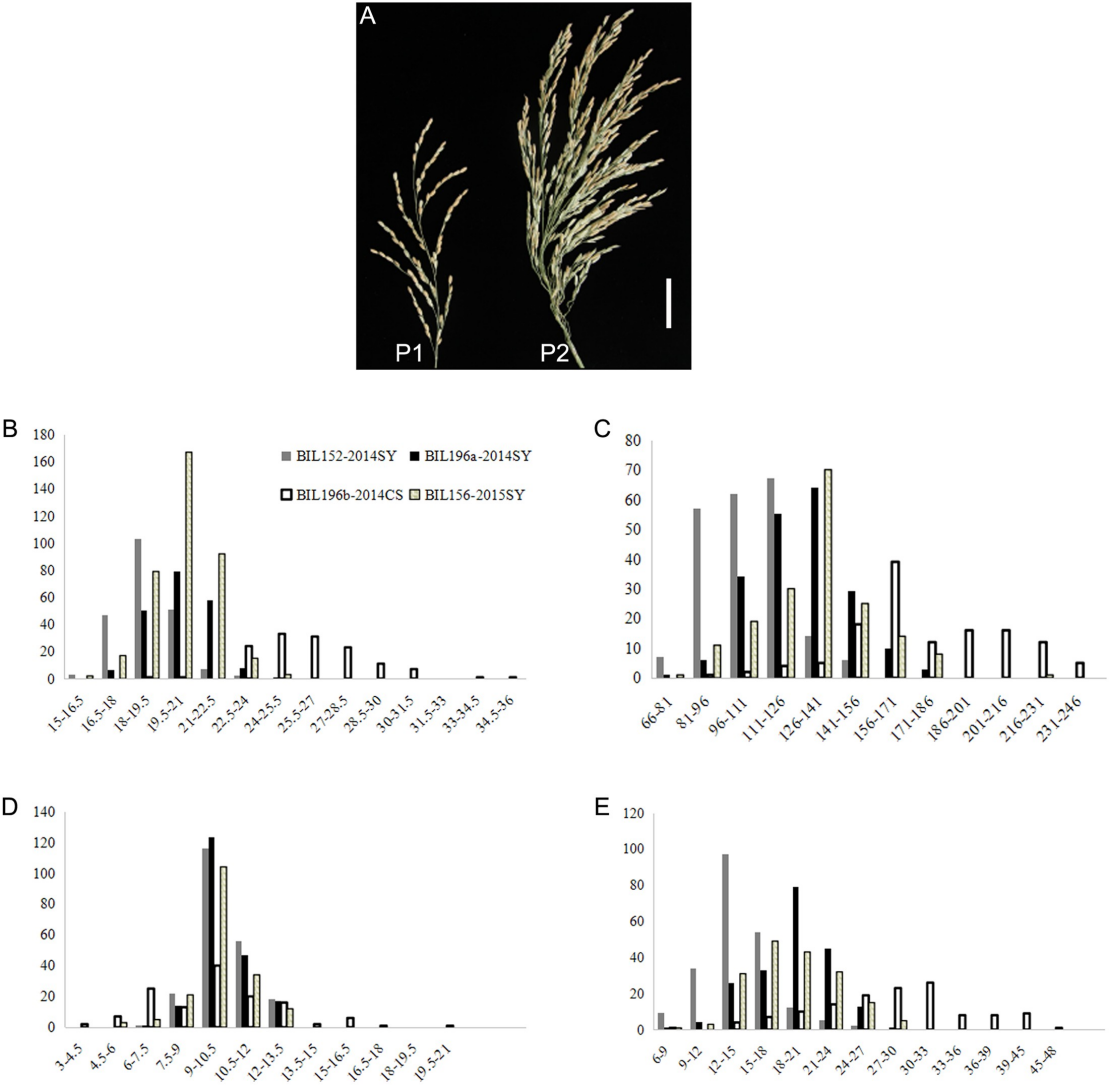
Field performance comparison between parents and distribution of yield components in the backcross populations. (A) Panicle shape comparison between R1128 and Nipponbare. The P1 and P2 represent Nipponbare and HR1128 respectively. Scale bar: 5 cm. (B) Panicle length. (C) Grain number per panicle. (D) Primary branch number. (E) Secondary branch number.

Notable differences were observed between the measured traits in Nipponbare and HR1128 (Table 1 and Fig 3A). The male parent HR1128 had higher grain number (maximum GN of 532.6 at CS in 2014) compared with the female parent ‘Nipponbare’ (maximum GN of 119.3 at CS in 2014). A similar situation was observed for the three traits PBN, SBN, and PL. The donor HR1128 exhibited a large panicle architecture with a panicle length of 30.2 cm and highly dense branching (PBN = 31.5 and SBN = 96.7) at CS in 2014, and the tendency was the same in the environment at SY in 2014 and 2015. The female parent ‘Nipponbare’ displayed a typical small panicle type with short panicle length of 17.6 cm, reduced branch numbers (PBN = 8.7 and SBN = 9.8) at SY in 2015, and had the lowest trait values among the different environments. The large trait differences between the two parents provided an abundant source of trait variation for population development and QTL mapping.

**Table 1:** Descriptive statistics and frequency distributions of panicle related traits in populations and parents.

Traits	Environment	BIL152		BIL196a		BIL196b		BIL196b-156		Parents	
		Range	Mean ± SD	Range	Mean ± SD	Range	Mean ± SD	Range	Mean ± SD	Nipponbare	HR1128
GN	Sanya,2014	66.0–153.0	104.2±15.71	74.0–175.0	125.2±17.40					98.1	421.9
	Changsha,2014					95.0–236.0	170.4±31.72			119.3	532.6
	Sanya,2015							71.0–221.0	125.3±22.79	88.9	397.1
PL(cm)	Sanya,2014	15.9–22.3	18.7±1.25	17.2–24.5	20.1±1.32					18.0	25.1
	Changsha,2014					18.6–34.1	25.8±2.38			23.8	30.2
	Sanya,2015							15.8–24.5	20.1±1.37	17.6	23.8
PBN	Sanya,2014	7.0–13.0	10.0±1.16	7.0–13.0	10.0±1.09					8.2	23.3
	Changsha,2014					8.0–17.0	12.7±1.50			9.8	31.5
	Sanya,2015							6.0–13.0	9.6±1.29	8.7	25.1
SBN	Sanya,2014	6.0–24.0	13.4±3.29	7.0–27.0	17.8±3.40					10.3	77.2
	Changsha,2014					9.0–45.0	25.9±7.47			14.8	96.7
	Sanya,2015							6.0–28.0	17.9±3.94	9.8	71.5

The descriptive statistics and frequency distributions of panicle related traits in the BIL (backcross inbred line) populations are presented in Fig 3 and Table 1. A large variation in the BILs was observed for GN, PL, PBN, and SBN in 2014 and 2015. The phenotypic values were all found to be continuous with normal frequency distributions, and the values of population skewness and kurtosis were all <1. Transgressive segregation was also observed for most of the traits which indicated that panicle component traits are controlled by multiple QTLs. Both of the above features of the BIL populations implied that all BILs were suited for QTL mapping of panicle-related traits.

Phenotypic correlation coefficients for BIL152, BIL196a, BIL196b, and BIL196b-156 were calculated and the results are shown in Table 2. All traits were found to be correlative to each other in different populations. For example, striking positive correlations were found between SBN and other panicle traits GN, PL and PBN in the BIL152 population. Similar phenomena were observed in all other populations. It was noteworthy that the correlation coefficients among panicle traits in BIL196b-156 were generally higher than those in other populations. the biggest and smallest correlation coefficients were all found between the same traits, PL and PBN; their values were 0.992 and 0.262 in BIL196b-156 and BIL196b respectively, and they were all significantly positively correlated at the 0.01 level.

**Table 2:** Correlation coefficients among four panicle traits, GN, PL, PBN and SBN for BIL152 (upper), BIL196a (secondary), BIL196b (tertiary) and BIL196b-156 (lower) populations.

	GN	PL	PBN
SBN	0.874**	0.596**	0.481**
	0.918**	0.692**	0.538**
	0.905**	0.586**	0.479**
	0.872**	0.966**	0.968**
PBN	0.687**	0.486**	
	0.655**	0.585**	
	0.589**	0.262**	
	0.776**	0.992**	
PL	0.654**		
	0.748**		
	0.491**		
	0.769**		

** is significant at p < 0.01.

### QTLs for panicle traits in the BILs

Three populations, BIL152, BIL196a, and BIL196b, were used for QTL analysis of panicle-related traits, and the results are shown in Table 3.

**Table 3:** QTL analysis of panicle related traits in populations.

Population	Environment	Trait Name	QTL Name	Position (cM)	Left Marker	Right Marker	LOD	PVE (%)	Add	Dom
BIL152	Sanya,2014	GN	*GN6-1*	109.00	RM7213	RM19962	4.15	11.08	-7.09	-4.04
		PL	*PL6-1*	121.00	RM20118	RM20210	5.71	12.43	-0.75	0.16
		PBN	*PBN6-1*	106.00	RM7213	RM19962	4.70	12.42	-0.53	-0.35
		SBN	*SBN6-1*	110.00	RM7213	RM19962	3.07	8.04	-1.36	-0.43
BIL196a	Sanya,2014	GN	*GN6-2*	6.00	RM20118	RM20210	4.63	10.66	-7.11	-1.53
		PL	*PL6-2*	6.00	RM20118	RM20210	5.98	12.69	-0.60	-0.08
		PL	*PL6-3*	65.00	RM412	RM20595	2.98	6.12	-0.10	-0.54
		PBN	*PBN6-2*	0.00	RM20000	RM6818	5.69	12.34	-0.45	-0.29
		SBN	*SBN6-2*	6.00	RM20118	RM20210	2.63	6.18	-1.04	-0.40
BIL196b	Changsha,2014	PL	*PL6-4*	40.00	RM20210	RM20018	3.06	12.67	-1.22	0.40
		SBN	*SBN6-3*	16.00	RM20000	RM20210	2.77	17.18	-3.20	-3.95
BIL196b-156	Sanya,2015	PL	*PL6-5*	3.00	RM20118	RM003	2.54	3.10	-0.26	-0.31

In BIL152, four QTLs for GN, PL, PBN, and SBN were detected on chromosome 6 (S1 Fig). QTL *PL6-1* had the highest LOD value (LOD = 5.71) and explained 12.43% of the phenotypic variation for panicle length among the all four genetic regions, and the HR1128 allele had an increase in PL. The other three QTLs, *GN6-1*, *PBN6-1*, and *SBN6-1*, shared the same interval between RM7213 and RM19962 with LOD values of 4.15, 4.70, and 3.07 and explained 11.08%, 12.42%, and 8.04% of phenotypic variations, respectively. The positive alleles of QTLs *GN6-1*, *PBN6-1*, and *SBN6-1* were all from the donor parent HR1128, and the *GN6-1* allele with the biggest additive effect could increase the grain number by 7.09.

Five QTLs for the four traits GN, PL, PBN, and SBN were identified in the BIL196a population at Shanya in 2014. The three QTLs for traits GN, PL, and SBN, *GN6-2*, *PL6-2*, and *SBN6-2*, were detected in the same chromosomal interval at the same position (S2 Fig). Among these, the QTL *PL6-2* had the highest LOD score (5.98) and explained the largest percentage of phenotypic variation (12.69%), similar to *PL6-1* detected in the BIL152 population at Shanya in 2014. All three QTLs had an increasing effect on their traits, and the positive alleles were from the male parent HR1128 as well. In addition, two other QTLs, *PL6-3* and *PBN6-2*, were also detected in this population in different genomic regions, and they had LOD values of 2.98 and 5.69, respectively. These two QTLs had a positive impact on PL and PBN, explaining 6.12% and 12.34% of the phenotypic variation separately, and the positive alleles came from the same parent, HR1128.

In the BIL196b population, there were only two QTLs for PL and SBN detected at Changsha in 2014 (S3 Fig). *PL6-4* with the higher LOD value (3.06) was mapped to the interval between markers RM20210 and RM20018, and explained 12.67% of the phenotypic variation explanation (PVE) for panicle length. The other QTL, *SBN6-3*, had a smaller LOD score (2.77) and was located between marker loci RM20000 and RM20210, but had larger phenotypic variation ratio (17.18%). It is noteworthy that the two QTLs mapped to nearly the same region on chromosome 6 as determined by the marker loci RM20000 and RM20018, and they both had a positive additive effect, with the positive alleles originating from the donor HR1128.

### Validation of the QTL for panicle length between marker loci RM20118 and RM20210

A segregating population, BIL196b-156, which consisted of 384 individuals, was developed to validate the genetic interval RM20118 to RM20210 for the panicle length QTL. As shown in Table 3 and S4 Fig, the QTL *PL6-5*, with LOD value 2.54 and 3.10% PVE, was detected in this population at Shanya in 2015. The QTL region was from marker loci RM20118 to RM003, which was located between marker loci RM20118 and RM20210. The genetic interval containing *PL6-5* was narrowed down to a physical region of 1.3 Mb on chromosome 6 that was smaller than the range for QTLs *PL6-1*, *PL6-2*, and *PL6-4*. The positive allele of *PL6-5* came from the cultivated variety HR1128 and it had an increasing effect on panicle length.

## Discussion

### QTL detection in advanced backcross overlapping rice populations

Panicle-related traits in rice include GN per panicle, PL, PBN, and SBN. All are typical quantitative traits that are controlled by major and minor genes and influenced by genetic background and the external environment. Because of these confounding factors, it is necessary to detect the QTLs in different environments and genetic backgrounds in order to validate the existence of actual QTLs. Comparison of QTLs detected in advanced backcross and overlapping populations of defined pedigree could improve the reliability of QTL detection [40–43]. In this research, four advanced backcross and overlapping populations (BIL152, BIL196a, BIL196b and BIL196b-156) were developed for the detection of panicle trait QTLs at Changsha and Sanya in 2014 and 2015. For example, the panicle length QTL *PL6-1*, which was detected in population BIL152, and *PL6-2*, which was detected in the BIL196a population, could support the existence and effects of each other, because the population BIL152 contained nearly all the heterozygous segments of chromosome 6 which were partially duplicated compared with BIL196a, and the QTL interval was located in the overlapping region. Another fact may certify the QTL PL6-1 could be detected in different environments. Populations BIL196a and BIL196b, two parts of the same segregating population, and BIL196b-156, which was derived from BIL196b, were grown at Sanya, Changsha, and Sanya in 2014, 2014, and 2015 respectively. The data obtained from two regions and three seasons gave strong support to the notion that the panicle length QTLs *PL6-2*, *PL6-4*, and *PL6-5* are the same, and that *PL6-2* had the highest LOD value of 5.98 along with the maximum 12.69% PVE.

### Comparison and analysis of QTLs

Twelve QTLs for the four panicle components GN, PL, PBN, and SBN were detected on chromosome 6 in this study. Based on the QTL physical positions, the QTLs were tightly linked with three chromosomal regions described as RM7213 to RM19962, RM20000 to RM20210, and RM412 to RM20595 (Table 4).

**Table 4:** Comparison and analysis of QTLs for panicle traits in three genetic regions.

Genetic regions	QTLs detected in this study	Common regions shared with previous studies
RM7213--RM19962	*GN6-1*	*qSPN-6* [44]; *qSPN-6* [45]; *gp6* [46]; *qgn6-3* [28]
	*PBN6-1*	N/A
	*SBN6-1*	*qsbn6-2* [28]
RM20000--RM20210	*GN6-2*	*gp6* [46]
	*PL6-1*,*PL6-2*,*PL6-4*,*PL6-5*	N/A
	*PBN6-2*	N/A
	*SBN6-2*,*SBN6-3*	*qsbn6-3* [28]
RM412--RM20595	*PL6-3*	*RM6811* [16]

Three QTLs, *GN6-1*, *PBN6-1*, and *SBN6-1*, were mapped to the first interval and they were all from the same population, BIL152, grown at Sanya in 2014. Compared with the results of previous studies, *qSPN-6* [44], *qSPN-6* [45], *gp6* [46], and *qgn6-3* [28] for grain number shared the common regions with *GN6-1* detected in this study. Among the four QTLs, *qSPN-6* and *gp6* had much larger intervals than *GN6-1* with LOD values of 3.0 and 6.7, and explained variance of 15.3% and 7.9%, respectively. The QTL *qgn6-3* mapped to the smallest physical region (0.4 cM), and was detected in our previous research using an F_2_ population derived from the same cross between HR1128 and ‘Nipponbare’, and it had a high LOD score (LOD = 15.62) and normal 8.9% PVE compared to *GN6-1*. The other QTL, *qsbn6-2* [28], located in the same region as *SBN6-1* and the *PBN6-1*, was not available from previous research, and it may be a novel QTL for primary branch number. Further studies should be focused on the grain number QTL *GN6-1* in consideration of the indirect verification from previous studies.

Eight QTLs, including *GN6-2*, *PL6-1*, *PL6-2*, *PL6-4*, *PL6-5*, *PBN6-2*, *SBN6-2*, and *SBN6-3*, mapped to the same chromosomal interval between marker loci RM20000 and RM20210. Among these, *GN6-2* was found in the area of *gp6* [46], *SBN6-2* and *SBN6-3* shared the overlapping interval that contained *qsbn6-3* [28], and originated from the same donor, HR1128. *PL6-1*, *PL6-2*, and *PL6-4* were detected in three different populations in two environments, which strongly supported this QTL having a stable genetic effect, and the validation of *PL6-5* provided further proof that all four panicle-length QTLs could be the same, with the highest LOD score of 5.98 and maximum PVE of 12.69%. It is noteworthy that four panicle-length QTLs (*PL6-1*, *PL6-2*, *PL6-4*, and *PL6-5*) and one branch number QTL 9*PBN6-2*) were not identified in previous studies, implying that they may be novel.

In the third chromosomal interval between marker loci RM412 and RM20595, only one panicle-length QTL, *PL6-3*, was mapped to this region which contains a previously mapped PL QTL linked to the SSR marker RM6811 [16]. In this study, a population consisting of 540 rice accessions were used for association mapping of panicle length. MLM (mixed linear model) analysis showed that SSR marker RM6811 on chromosome 6 was associated with panicle length (p < 0.05) in 2011 and 2012. The QTL PVE ranged from 3.96 to 4.51% compared to *PL6-3*, where the PVE was 6.12%.

### QTL detection in the pericentromeric region

**Fig 4:**
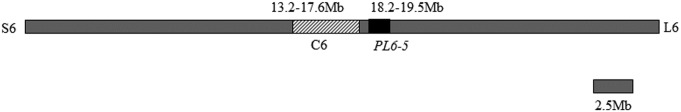
Map position of QTL *PL6-5* in the pericentromeric region on chromosome 6. The abbreviations S6 and L6 denote the short arm and long arm region of chromosome 6 respectively. C6 indicates the centromere region from 13.2 Mb to 17.6 Mb. The physical position of QTL *PL6-5* was mapped between 18.2 Mb and 19.5 Mb and this region is close to the centromere, separated by only 600 kb.

Although map-based cloning has been used successfully in the dissection and identification of many complex quantitative traits in rice, several obstacles, such as depression of meiotic recombination in the centromeric region, can make map-based cloning very difficult [47]. The important yield gene *Ghd7* was initially fine mapped between marker loci RM5436 and C39 in the centromeric region on chromosome 7, and severe recombination suppression was observed in the targeted interval which complicated cloning of the gene. To solve this problem, the candidate gene method was used, which resulted in the cloning of *Ghd7* [48]. Some studies have implied that depression of meiotic recombination occurs not only around the centromere, but could also be present in the pericentromeric region. One putative QTL, *qSPP7*, which controls the number of spikelets per panicle, was identified in the pericentromeric region of rice chromosome 7. In order to isolate the QTL, 1,082 plants with extremely small panicles from a BC_3_F_2_ population containing 8,400 individuals were further used to fine map the QTL. This showed that *qSPP7* co-segregated with two marker loci, RM5436 and RM5499, that spanned a physical distance of 912.4 kb. Overall, these results suggested that recombination suppression occurs in the region, and that a positional cloning strategy is not feasible for isolating *qSPP7* [49]. A similar phenomenon may have occurred in our study. The panicle length QTLs *PL6-1*, *PL6-2*, and *PL6-4* were initially mapped to the same region between marker loci RM20118 and RM20210, and a larger population, BIL196b-156 (BC_3_F_4_), consisting of 385 individuals, was then used to narrow down the *PL6-5* locus to a physical interval of 1.3 Mb between RM20118 and RM003. We found it strange that the LOD score and PVE in the BIL196b-156 (LOD = 2.54, PVE = 3.1%) were much smaller than in the other three populations. Possible explanations include the occurrence of severe recombination suppression in the BIL196b-156 population, because the RM20118 locus is close to the pericentromeric region of rice chromosome 6 (Fig 4), or that the QTL effect was altered in the different environments under various genetic backgrounds. However, existence of the QTL *PL6-5*, that has a significant effect on panicle length in rice, is supported by the fact that it was detected by QTL analyses in four populations. Strategies such as large-scale NIL (near-isogenic line) and F_2_ segregating populations combined with bioinformatics prediction may be required for the isolation and cloning of *PL6-5*.

## Supporting information

S1 Fig Quantitative trait locus analysis of panicle traits using BIL152 individuals. (TIF)

S2 Fig Quantitative trait locus analysis of panicle traits using BIL196a individuals. (TIF)

S3 Fig Quantitative trait locus analysis of panicle traits using BIL196b individuals. (TIF)

S4 Fig Quantitative trait locus analysis of panicle traits using BIL196b-156 individuals. (TIF)
