# Supplementary figures and images for: QTL analysis and dissection of panicle components in rice using advanced backcross populations derived from Oryza Sativa cultivars HR1128 and ‘Nipponbare’
Source: PLoS One. 2017 Apr 19;12(4):e0175692. doi: 10.1371/journal.pone.0175692 (PMC5396889; doi:10.1371/journal.pone.0175692)

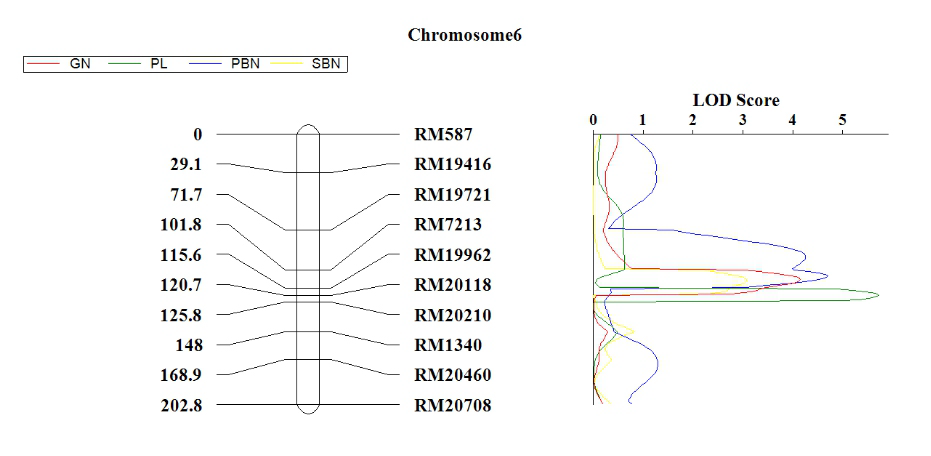

Supplement: S1 Fig — (TIF) [file pone.0175692.s001.tif]

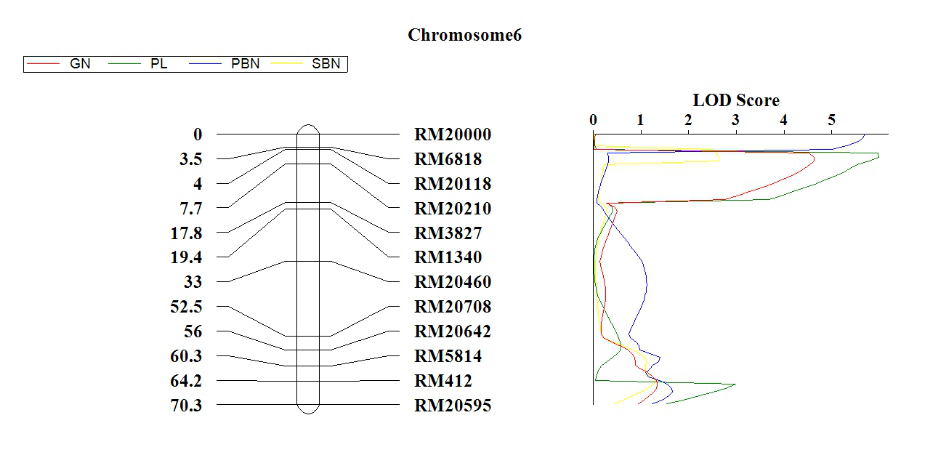

Supplement: S2 Fig — (TIF) [file pone.0175692.s002.tif]

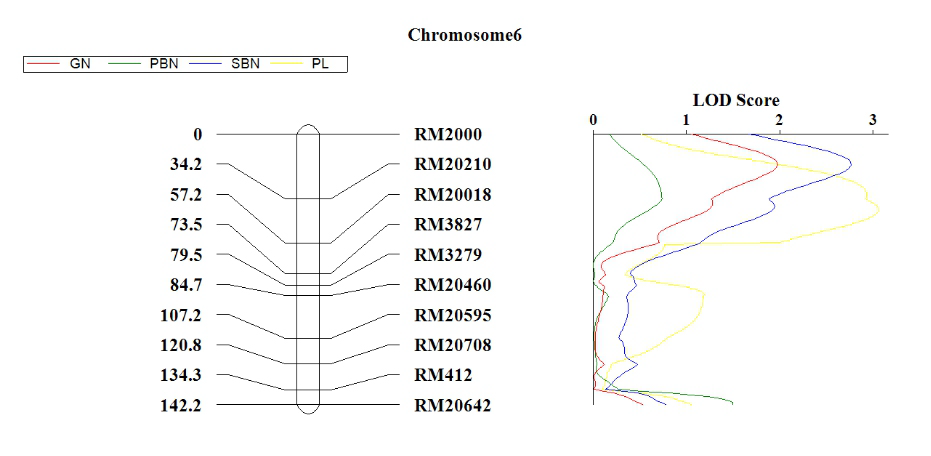

Supplement: S3 Fig — (TIF) [file pone.0175692.s003.tif]

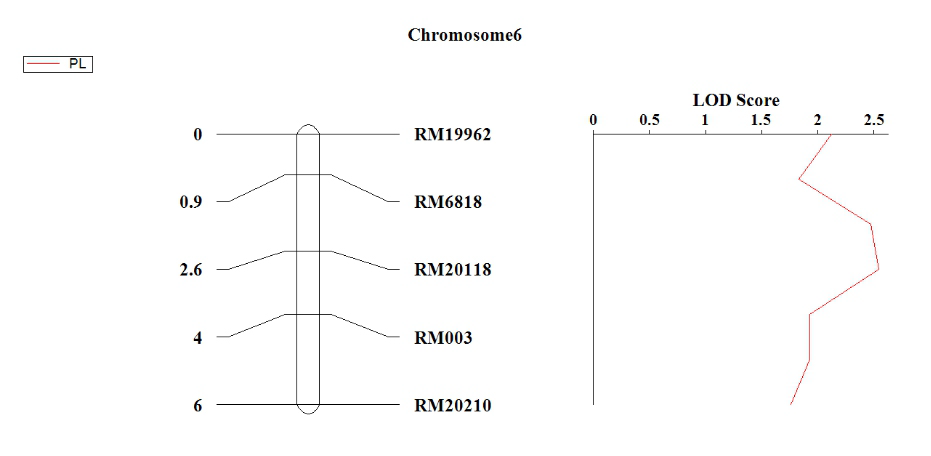

Supplement: S4 Fig — (TIF) [file pone.0175692.s004.tif]
